# Magnitude and Predictors of In-Hospital Stroke Mortality in Ethiopia: A Systematic Review and Meta-Analysis

**DOI:** 10.1155/2022/7202657

**Published:** 2022-05-24

**Authors:** Amare Abera Tareke, Masrie Getnet Abate, Addis Alem, Yoseph Merkeb Alamneh, Alehegn Aderaw Alamneh, Yikeber Argachew Deml, Mulu Shiferaw, Woldeteklehaymanot Kassahun, Abraham Teym

**Affiliations:** ^1^Physiology Unit, Department of Biomedical Sciences, College of Medicine and Health Sciences, Wollo University, Dessie, Ethiopia; ^2^Biostatistics Unit, Department of Epidemiology, Faculty of Public Health, Jimma University, Jimma, Ethiopia; ^3^Biochemistry Unit, Department of Biomedical Sciences, College of Medicine and Health Sciences, Wollo University, Dessie, Ethiopia; ^4^Department of Biomedical Sciences, School of Medicine, Debre Markos University, Debre Markos, Ethiopia; ^5^Department of Human Nutrition, College of Health Science, Debre Markos University, Debre Markos, Ethiopia; ^6^Biomedical Team, School of Nursing, College of Health Sciences, Woldia University, Woldia, Ethiopia; ^7^Department of Medical Laboratory Sciences, College of Health Sciences, Woldia University, Woldia, Ethiopia; ^8^Department of Environmental Health, College of Health Sciences, Debre Markos University, Debre Markos, Ethiopia

## Abstract

**Introduction:**

Stroke is the second leading cause of mortality worldwide, accounting for approximately 5.5 million deaths each year. Due to demographic and health changes, the epidemiology of stroke is shifting from industrialized to low- and middle-income nations. Ethiopia is a developing country with a population that reflects this shift. Therefore, this systematic review and meta-analysis are aimed at evaluating the extent of in-hospital mortality of both ischemic and hemorrhagic stroke in Ethiopia and determining relevant factors associated with the mortality.

**Methods:**

Observational studies published as of July 15, 2020, that reported the magnitude, predictors, and causes of in-hospital mortality of stroke were systematically and comprehensively retrieved using the PRISMA 2020 criteria from databases such as PubMed/MEDLINE, Science Direct, and Google Scholar. The review papers were chosen based on the study methodology (facility-based observational), the study area (Ethiopia), the study population (adult patients with stroke), the outcome (in-hospital mortality), and the fact that they were published in English.

**Result:**

A total of 3709 patients with stroke were included in this systematic review and meta-analysis, which included 19 publications. In-hospital mortality was 14.03 percent on average in the studies, with reports ranging from 6.04 percent to 37.37 percent. Patients with hemorrhagic type stroke, admission Glasgow Coma Scale less than or equal to 12, impaired mental status, National Institutes of Health Stroke Scale stroke level greater than 13, prolonged hospital stay, any incontinence, pneumonia, and/or swallowing trouble had an increased risk of death after stroke.

**Conclusion:**

The magnitude of in-hospital mortality of patients with stroke in Ethiopia is high. The assessment of the level of consciousness is vital for clinical management and as an indicator of prognosis. Patients with unfavorable prognostic signs, such as entry Glasgow Coma Scale, National Institutes of Health Stroke Scale stroke level > 13, hemorrhagic stroke, pneumonia, incontinence, and dysphagia, should be given priority.

## 1. Introduction

Globally, the burden of stroke has increased substantially over the past few decades partly due to the rising population and higher number of aged individuals [1]. The increase in modifiable risk factors especially in low- and middle-income countries (LMICs) predicts the persistent growth of the number of patients who will need care by clinicians with expertise in neurological conditions [2]. According to the Global Burden of Disease 2016 report, the estimated lifetime risk of stroke in 2016 for those aged 25 years or older was 24.9%, showing an increment from 1990 to 22.8% [3]. In 2016, 5.5 million deaths and 116.4 million disability-adjusted life years occurred due to stroke word wide [4].

The highest stroke-related death was registered in Africa at 29% with a 95% confidence interval of 23% to 36% [5]. Africa has a slightly greater preponderance of small vessel disease-related stroke and intracerebral haemorrhagic lesions than elsewhere in the world [6]. Even though hypertension remained the most important modifiable risk factor, other risk factors include diabetes mellitus, dyslipidemia, obesity, stress, smoking, alcohol use, physical inactivity, and an unhealthy diet. Patients in LMICs more often have severe strokes, intracerebral hemorrhage, poorer access to services, and used fewer investigations and treatments than those in high-income countries. These differences in patient characteristics can explain the poorer clinical outcomes [7]. In Ethiopia, 32860 deaths occurred in 2016 because of stroke. This figure has a 23.8% increment from deaths that occurred in 2000 [8]. In-hospital mortality in Ethiopia ranges from 6.04% to 37.37% in different parts of the country.

Several factors were listed as predictors of mortality elsewhere. Older age, male sex, educational level, loss of consciousness, lower Glasgow Coma Scale (GCS) score, higher National Institutes of Health Stroke Scale (NIHSS) score, comorbidities, complications, systolic blood pressure, severe diastolic pressure, second or more episode of stroke, seizures, abnormal pupillary size, hemorrhagic stroke type, presence of aspiration pneumonitis, random blood sugar (RBS) > 200 mg/dl, and neutrophil-to-lymphocyte ratio were independent predictors of mortality in stroke [9–11]. Aspiration pneumonia, septicemia, brain hernia, circulatory and respiratory failure, cardiac arrest, sudden death, heart failure, and multiorgan failure were among the causes of death in patients admitted with stroke [12, 13]. These peculiar factors are also responsible for the substantial disparities in incidence velocity, ischemic stroke proportion, and case fatality status in LMICs, which remains unknown [14].

To tackle the burden of neurological disorders like stroke reliable data on epidemiology, risk factors, mortality, prevention, care delivery, care integration, and community perception are urgently needed [15]. Despite many studies conducted to estimate the magnitude of in-hospital mortality, predictors, and possible causes, a nationally representative study is yet to be conducted. A recent meta-analysis [16] is conducted in Ethiopia that reports risk factors and in-hospital mortality. The current study has comprehensive search results for in-hospital mortality. Besides, it showed predictors of stroke-related mortality. The present study was performed to pool the burden of in-hospital stroke mortality and determine the predictors of mortality in Ethiopia.

## 2. Methods

### 2.1. Literature Search

Observational studies reporting in-hospital mortality, predictors, and causes in Ethiopia were systematically and comprehensibly searched using the following databases PubMed, ScienceDirect, and Google Scholar according to the preferred reporting items for systematic reviews and meta-analysis (Supplementary file 1) [17]. All published original articles with data on risk factors and/or clinical presentation of patients with stroke in Ethiopia up to July 15, 2020, were included in this study. The articles were searched using search terms; (stroke[MeSH Terms] OR stroke OR “brain infarction” OR “cerebral infarction” OR “cerebrovascular accident” OR “ischemic stroke” OR “hemorrhagic stroke”) AND (determinants OR “associated factors” OR predictors) AND (outcome OR mortality) AND (Ethiopia) NOT “South Africa” NOT Africa NOT Egypt NOT Nigeria NOT Ghana NOT Kenya NOT “Meta-analysis” NOT “systematic review” NOT Abstract) NOT global. A manual search of references from included studies was also conducted.

### 2.2. Study Selection

The following inclusion and exclusion criteria were applied in determining study eligibility. Studies with facility-based observational design conducted on adult patients with stroke reporting in-hospital stroke mortality in Ethiopia were included. In addition, the study included articles published in English language only.

Studies which report stroke outcome as “poor” and “good” without the actual figure or percentage of deaths were excluded. Determinants of stroke outcome, with “poor” and “good” outcomes, were not included in this meta-analysis as well. This meta-analysis does not include review articles, incomplete articles, conference proceedings, and duplicates. All the above-mentioned designed researches conducted on adults and as of July 15, 2020, were included. Two authors (AAT and MGA) independently screened the titles, abstracts, and full-text of retrieved articles to identify their eligibility, and disagreements were solved by discussion and consensus.

### 2.3. Data Extraction

The data extraction was performed by two independent authors (AAT, AT) using Microsoft Excel. Data extraction included first author, publication year, design of the study, sample size, participants' sex, the region of the study/study area, mean age, mortality rate, different mortality predictors, and causes of death.

### 2.4. Outcome Measurement and Statistical Analysis

The magnitude of in-hospital stroke mortality was pooled with a 95% confidence interval (CI) using the random-effects, inverse variance method. The causes of mortality were pooled with the same method. The odds ratio (OR) with 95% CI of possible predictors was also pooled after defining variables for easiness of analysis. Subgroup analysis was performed. Statistical heterogeneity was measured by *I*^2^ statistic and we consider *I*^2^ > 50% as notable heterogeneity [18].

For statistical feasibility, we dichotomized predictors. The admission GCS was classified into two, less than or equal to 12 and greater than 12. The mental status of patients with stroke is dichotomized into altered and normal. The altered category encompasses loss of consciousness, coma, and others. NIHSS is again classified into two ≤13 and >13. Hospital length of stay is divided into less than or equal to 10 and greater than 10. Incontinence represents any form of it.

Potential publication bias was assessed using a funnel plot. The risk of bias of the included studies were assessed using the Newcastle Ottawa quality assessment scale for cross sectional studies. To detect the robustness of the results, a sensitivity analysis was conducted by sequential elimination of each study from the pool. *P* value ≤ 0.05 cut-point was used to declare statistical significance. All analyses were performed using STATA software (Version 16, StataCorp, Texas, USA).

## 3. Results

### 3.1. Search Results and Characteristics of Included Studies

**Figure 1:**
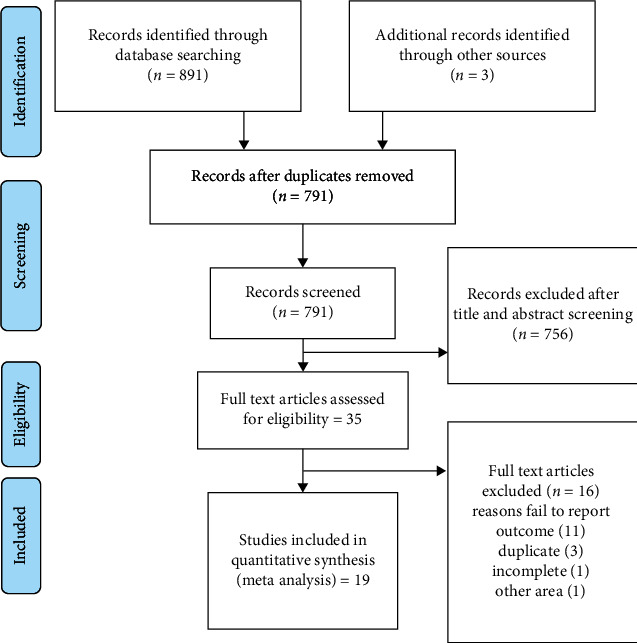
Literature search results and study selection.

A total of 894 articles were generated. Of which, 35 articles were fully screened. Finally, nineteen studies with a total of 3709 patients with stroke were eligible for this systematic review and meta-analysis (Figure 1).

Only four of the 19 studies were prospective, with the majority being retrospective cross-sectional studies. The geographical distribution of the studies revealed that Addis Ababa and Amhara shared the majority of the studies, with six studies each. The Oromia region had three studies, while the rest of Ethiopian regions had three. In various studies, mortality ranged from 6.04 percent to 37.37 percent. A variety of factors were identified as predictors and causes of death (Table 1).

### 3.2. Magnitude of Stroke In-Hospital Mortality

**Figure 2:**
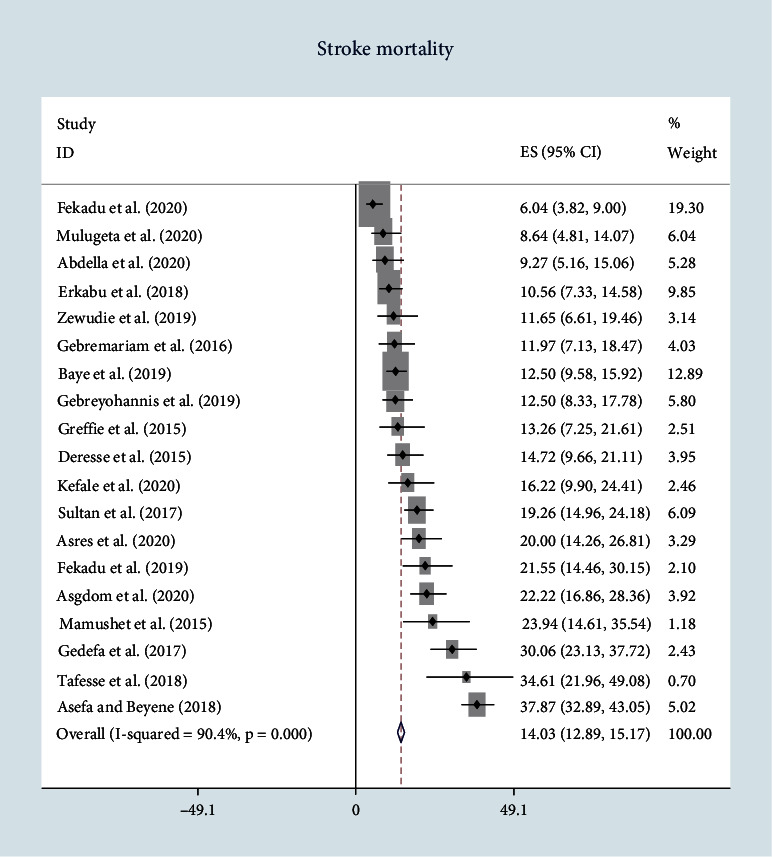
Forest plot of pooled in-hospital stroke mortality.

The magnitude of in-hospital mortality was 14.03 with 95% CI [12.89-15.17] *I*^2^ = 90.4% (Figure 2). The lowest mortality rate was reported by Fekadu et al. 2020 with in-hospital mortality of 6.04%, and the highest mortality was recorded by Asefa and Beyene 2018 with prevalence of 37.87% (Figure 2).

**Figure 3:**
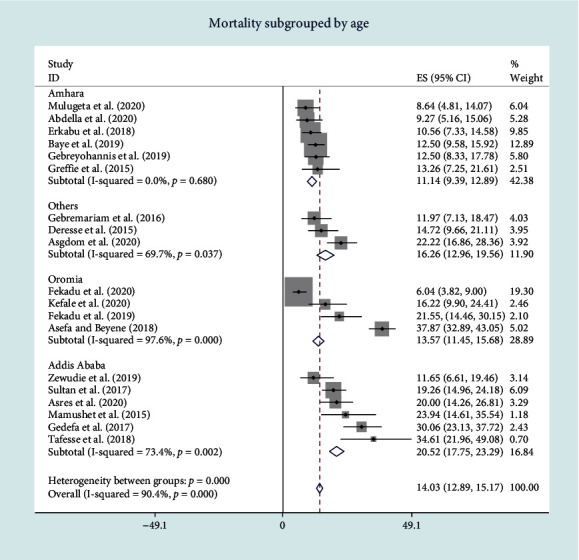
Forest plot of pooled in-hospital stroke mortality subgrouped by geographical regions.

The subgroup analysis based on the study area (region in this case) indicated the presence of disparities in mortality rate. In-hospital stroke mortality in the Capital-Addis Ababa was the highest [mortality rate = 20.52%, 95% CI: 17.75-23.29, and *I*^2^ = 73.4%]. Amhara region has lowest in-hospital stroke mortality [mortality rate = 11.14, 95% CI: 9.39-12.89, and *I*^2^ = 0.0%] (Figure 3).

**Figure 4:**
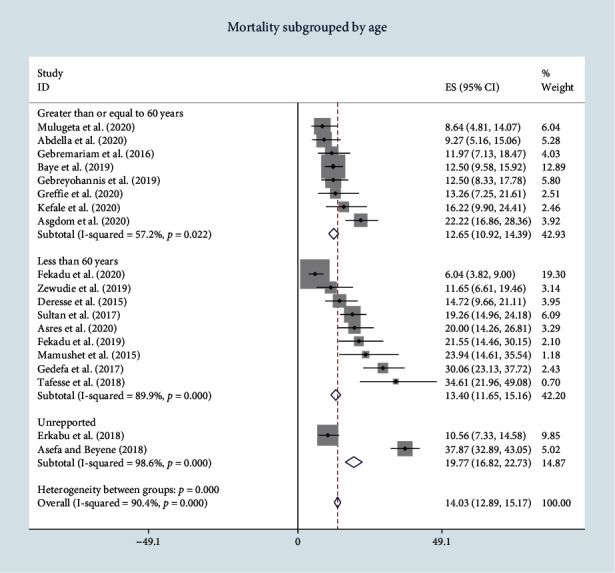
Forest plot of pooled in-hospital stroke mortality subgrouped by age.

Seventeen studies reported age of participants, from which the age was categorized as less than and greater than equal to 60. Two studies fail to report patient's age and fall under the category of unreported. The subgroup analysis of in-hospital mortality based on patient's age shows that mortality was higher in patients of whose mean age less than 60 years as compared to those who were older (Figure 4).

### 3.3. Risk Factors

**Figure 5:**
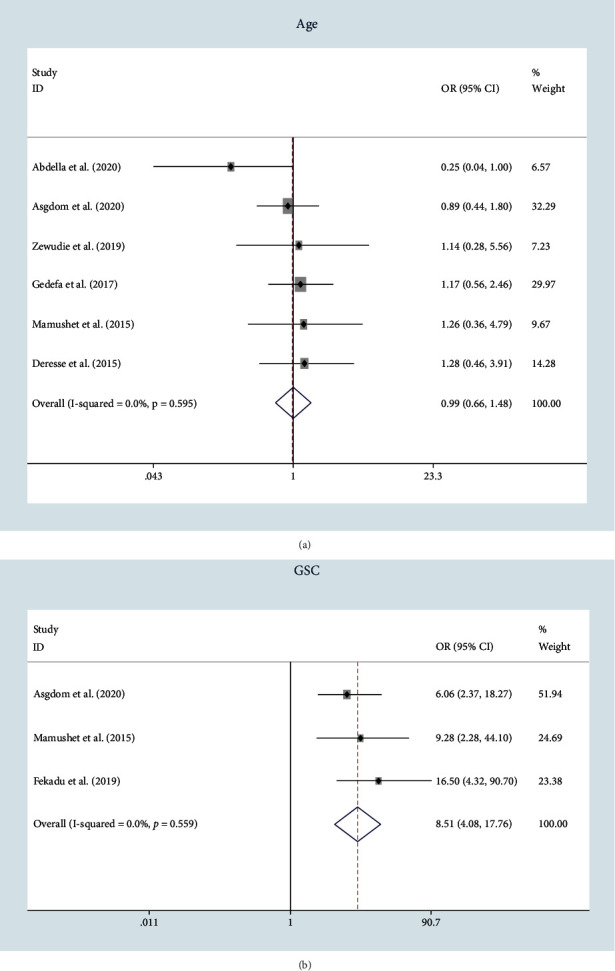
(a) Forest plot showing the odds of stroke mortality by sex. (b) Forest plot showing the odds of stroke mortality by GCS.

Eleven risk factors were identified. There was no significant difference in stroke mortality among males and females (Figure 5(a)). The type of stroke had determinant effect on stroke outcome. Patients with hemorrhagic stroke type had higher odds of death with OR = 1.81; 95% CI: (1.13–2.92), *P* value = 0.015, and *I*^2^ = 58.2%. Admission GCS and mental status were also strong predictors of death (Figure 5(b)). GCS during admission less than or equal to 12 and altered mental status have odds of 8.51 and 5.59, respectively, compared with GCS greater than 12 and normal mental status. NIHSS, hospital length of stay, occurrence of any incontinence, pneumonia, and swallowing difficulty were other predictors of death in admitted patients with stroke in Ethiopia (Table 2).

## 4. Discussion

This study provided the pooled result of stroke mortality in Ethiopian hospitals. It also synthesizes evidence on the predictors of mortality from available literature.

The pooled prevalence of in-hospital stroke mortality was found to be 14.03%. This finding was in line with the result of systematic reviews and meta-analyses conducted in Ethiopia and Eastern African countries with a mortality rates of 18% and 15%, respectively [16, 19]. But it was higher than a study conducted in Argentina (2.5%) [20]. This disparity could be due to low access to health facilities for routine medical checkup which could prevent the adverse outcomes of stroke and inadequate infrastructure and trained personnel with low level of care given in those established facilities in Ethiopia. The magnitude of in-hospital stroke mortality in Ethiopia was lower than a finding from a systematic review of sub-Saharan Africa of pooled mortality rate (22%) and studies reported from Nigeria, Kenya, Burkina Faso, Uganda, and Madagascar with mortality rates of 35%, 21.7%, 28.7%, 31%, and 30%, respectively [11, 21–24]. The difference could be explained by variations in the rate of patient with stroke admissions in hospitals.

The subgroup analysis by region showed that the magnitude of in-hospital mortality of stroke was higher in the capital Addis Ababa (20.56%) than Amhara (11.14%), Oromia (13.57%), and others (16.56%). This may be related to the effect of urbanization, access to health facility, and health seeking behavior differences, as those who live in urban areas are able to get health facilities and have greater health seeking behavior. This may increase the rate of admission to hospitals, and the numbers of case fatalities became higher in Addis Ababa, whereas rural residents may not visit health facilities and in-hospital mortality of stroke become decreased [25–28], and stroke mortality in rural areas might be under reported.

Cognizant with the current meta-analysis, previous studies reported the severity of stroke was determinant in survival of patients with stroke [29]. Also, studies conducted in Tanzania, Uganda, and Nigeria [11, 23, 30] underpinned the influence NIHSS in mortality rate of patients with stroke. These findings support out result of patients having NIHSS scores of greater than 13 at admission have greater odds of death.

The level of consciousness at admission is also key indicator of the degree of brain injury after stroke [31]. This concept is well supported by the current findings where lower admission GCS and altered mental status increased the odds of in-hospital mortality (OR = 8.51 and OR = 5.59, respectively. Besides, it was consistent with the systematic review and meta-analysis performed in the sub-Sharan Africa region, and other hospital-based studies in Uganda, Nigeria, and China [11, 19, 23, 32].

As the brain suffered from injuries like stroke, motor output from cortical and subcortical structure will be impaired. The oropharyngeal, gastrointestinal, and urinary tract muscles which are controlled by cortical and subcortical brain regions through cranial and spinal nerves will be involved as well [33, 34]. From this meta-analysis, patients with stroke with swallowing difficulty (OR = 4.73) and any incontinence (OR = 4.87) were found to have higher odds of in-hospital death than their counters. Swallowing difficulty poses the life of patients with stroke at risk of developing aspiration pneumonia, dehydration, and nutritional deficiency which facilitates the worst outcome-death [35]. The result of this study shows that swallowing difficulty was a predictor for in-hospital mortality, which was in-line with findings from USA, Switzerland, and UK [36–38]. In addition, patients having any incontinence had higher risk of in-hospital mortality, which was similar with the result from a systematic review and meta-analysis [39] and other prospective studies from Gambia and China [32, 40].

The development of infections such as aspiration pneumonia (OR = 4.43) was found as a potential predictor for in-hospital mortality which was in-line with findings from USA, Ireland, England, Thailand, Germany, Denmark, and China [32, 41–45]. Pneumonia causes the highest attributable mortality of all medical complications following stroke, and the risk of aspiration pneumonia was higher in age greater than 65 years, patients with aphasia, decreased cognition, and dysfunctional swallowing [46]. The length of hospital stay was found to be a significant predictor for in-hospital stroke mortality. While this does not mean that the length of hospital stay leads to death by itself rather as the patients' hospitalization increased, it will increase patients' exposure to infections like pneumonia and urinary tract infection as the stroke suppresses their immunity. On the other hand, the length of hospital stay might implicate the presence of neurological complication, and the development of neurological complications in stroke is also a key indicator of prolonged hospital stay [43, 45, 47–50]. Additionally, the severity of stroke and swallowing difficulty are stated as the predictors for prolonged hospital stay previously [44, 48]. These factors are also identified as the predictors of in-hospital stroke mortality in this study.

The type of stroke had a determinant effect on the patients' outcomes. Patients with hemorrhagic stroke type had higher odds of death (OR = 1.81) than ischemic stroke. The result of this study was in line with a finding from a systematic review in sub-Saharan African countries and single reports from Uganda, Denmark, and Japan [23, 51, 52]. The reason for this is that hemorrhagic stroke leads to more complicated brain damage as blood vessels rupture and bleed into the brain tissue which in turn builds up intracranial pressure, irritation, and swelling of the surrounding tissue while ischemic strokes result in localized or diffused cerebrovascular injury [53–56]. Hemorrhage also can increase intracranial pressure (ICP); elevated ICP can lead to death or devastating neurological damage either by reducing cerebral perfusion pressure (CPP) and causing cerebral ischemia or by compressing and causing hernia of the blood stream or other vital organ [57]. Similarly, aspiration pneumonia was identified to play the role of worsening admitted patient's outcome leading to death.

The findings of this study should be interpreted considering the following limitations. The high level of heterogeneity should be interpreted cautiously. Even though this study covered majority of the geographical areas of the country, most studies were concentrated on some regions of the country especially Amhara, Oromia, Addis Ababa, and Tigray regions. There are still poorly represented or unrepresented geographical areas, in which the inclusion of these areas might alter the finding of this study. Thus, lack of geographical representation appeared to be the limitation of this study.

## 5. Conclusions

The magnitude of in-hospital mortality of patients with stroke in Ethiopia is high. The assessment of level of consciousness is vital for clinical management and as an indicator of prognosis. Patients with unfavorable prognostic signs, such as entry GCS, NIHSS stroke level > 13, hemorrhagic stroke, pneumonia, incontinence, and dysphagia, should be given priority.

## Figures and Tables

**Table 1:** Characteristics of included studies.

	Author/year	Area	Design	Period	Sample size	M/F	Mean age (^∗^median)	Mortality	Factors reported	Causes of death
1	Abdella et al. 2020 [58]	Amhara	R	2015-2017	151	75/76	65^∗^	9.27	Aspiration pneumonia, admission swallow test, incontinence, mental status, increased ICP, sex, length of hospital stay	Increased ICP, sepsis, others
2	Asefa and Beyene [59]	Oromia	R	2015-2017	367	234/133	NR	37.37	Age, sex	NR
3	Asgedom et al. 2020 [60]	Tigray	R	2018-2019	216	90/126	61.2	22.22	Sex, age, region, residence, BP at admission cholesterol level, RBS, creatinine, potassium, EF, GCS, type of stroke, history of previous stroke, time to hospital arrival, comorbidity, medication at admission, antihypertensive drugs, statins use, anticoagulant use, hospital stay	NR
4	Asres et al. 2020 [61]	AA	R	2015-2018	170	97/73	52.49	20.0	NR	Aspiration pneumonia HF, increased ICP, ARDS
5	Baye et al. 2020 [62]	Amhara	R	2012-2018	448	188/260	63.9	12.5	NR	NR
6	Deresse et al. 2015 [63]	South	P	2013-2014	163	108/55	53.1	14.72	Sex, mental status, seizure, NIHSS, time from onset of stroke to admission, stroke types	Increased ICP, aspiration pneumonia, refractory status epilepticus
7	Erkabu et al. 2018 [64]	Amhara	R	2014-2016	303	191/112	NR	10.56	NR	NR
8	Fekadu et al. 2019 [65]	Oromia	P	2017	116	73/43	55.1	21.55	GCS, NIHSS, incontinence, mental status, means of stroke diagnosis, type of the stroke brain edema complication, swallowing difficulty, aspiration pneumonia complication, previous history of medication	Increased ICP, aspiration pneumonia, IHD, ICH, stroke, refractory status epileptics, hypertensive encephalopathy, renal/hepatic diseases, other
9	Fekadu et al. 2020 [66]	Oromia	R	2013-2017	364	208/156	59.66	6.04	NR	NR
10	Gebremariam et al. 2016 [67]	Tigray	R	2012-2014	142	77/65	62.8	11.97	NR	NR
11	Gebreyohannes et al. 2019 [68]	Amhara	R	2012-2016	208	88/120	65.17	12.5	Vascular disease, VHD, infection, hematocrit, SGOT, BUN, serum creatinine, GCS, statin use, warfarin use, hemorrhagic transformation	Aspiration pneumonia, HAP, UTI, bed sore, hemiplegia/hemiparesis
12	Gedefa et al. 2017 [69]	AA	R	2015-2016	163	92/71	57.5	30.06	NR	NR
13	Greffie et al. 2015 [70]	Amhara	R	2010-2013	98	46/52	68^∗^	13.26	NR	Aspiration pneumonia, increased ICP, unknown
14	Kefale et al. 2020 [71]	Oromia	R	2016-2019	111	55/56	63.36	16.22	NR	NR
15	Mamushet et al. 2015 [72]	AA	P	2008-2009	71	43/28	52.76	23.94	Age, sex, delay in admission, length of hospital stays, GCS, types of stroke, complications	NR
16	Mulugeta et al. 2020 [73]	Amhara	R	2017-2019	162	75/87	60^∗^	8.64	NR	NR
17	Sultan et al. 2017 [74]	AA	R	2010-2014	301	128/123	55	19.26	NR	NR
18	Mengesha 2018 [75]	AA	R	2001-2012	52	29/23	45.3	34.61	NR	NR
19	Zewde et al. 2019 [76]	AA	P	2015-2016	103	66/37	55.5	11.65	Age, gender, admission blood glucose level, type of stroke, NIHSS	NR

All the studies were cross sectional studies; the P and R in the design column represent the prospective and retrospective nature of these studies, respectively. The M/F column shows the number of males and female patients in the study. The age column described the mean age of participants, unless starred with ^∗^ which represents median age. AA: Addis Ababa; BUN: blood urea nitrogen; EF: ejection fraction; GCS: Glasgow Coma Scale; HAP: hospital-acquired pneumonia; HF: heart failure; ICH: intracranial hemorrhage; ICP: intracranial pressure; IHD: ischemic heart disease; NIHSS: National Institute of Health Stroke Scale; NR: not reported; RBS: random blood sugar; SGOT: serum glutamic oxaloacetic transaminase; UTI: urinary tract infections; VHD: valvular heart disease.

**Table 2:** Meta-analysis of risk factors for in-hospital stroke mortality in Ethiopia.

No.	Risk factor		Number of studies	Meta-analysis			Heterogeneity	
		Categories		OR	95% CI	*P* value	*I* ^2^	*P* value
1	Sex	Male^∗^ vs. female	6	0.99	[0.66–1.48]	0.957	0.0	0.595
2	Type of stroke	Ischemic^∗^ vs. hemorrhagic	5	1.81	[1.13–2.92]	0.015	58.2	0.048
3	GCS	>12^∗^ vs. ≤12	3	8.51	[4.08–17.76]	<0.001	0.0	0.559
4	Mental status	Normal^∗^ vs. altered	3	5.59	[3.87–23.05]	<0.001	0.0	0.488
5	Arrival time	>24^∗^ vs. ≤24	2	0.82	[0.37–1.80]	0.618	31.8	0.226
6	Medication	Yes^∗^ vs. no	2	0.70	[0.35–1.40]	0.308	51.3	0.152
7	NIHSS	≤13^∗^ vs. >13	2	16.07	[5.25–49.13]	<0.001	0.0	0.698
8	Length of stay		2	2.73	[1.23–6.02]	0.013	0.0	0.879
9	Incontinence	No^∗^ vs. yes	2	4.87	[1.96–11.12]	0.001	0.0	0.435
10	Pneumonia	No^∗^ vs. yes	2	4.43	[1.90–10.32]	0.001	0.0	0.678
11	Swallowing difficulty	No^∗^ vs. yes	2	4.73	[1.54–14.53]	0.007	0.0	0.588

^∗^Reference group. GCS: Glasgow Coma Scale; NIHSS: National Institute of Health Stroke Scale.

## Data Availability

The datasets analyzed during the current study are available from the corresponding author upon reasonable request.

## Supplementary Materials

Supplementary Materials Supplementary file 1: PRISMA 2020 checklist.
